# Functional connectivity directionality between large-scale resting-state networks across typical and non-typical trajectories in children and adolescence

**DOI:** 10.1371/journal.pone.0276221

**Published:** 2022-12-01

**Authors:** Martina J. Lund, Dag Alnæs, Jaroslav Rokicki, Simon Schwab, Ole A. Andreassen, Lars T. Westlye, Tobias Kaufmann

**Affiliations:** 1 Norwegian Centre for Mental Disorders Research (NORMENT), Division of Mental Health and Addiction, Oslo University Hospital and Institute of Clinical Medicine, University of Oslo, Oslo, Norway; 2 Bjørknes College, Oslo, Norway; 3 Department of Psychology, University of Oslo, Oslo, Norway; 4 Center for Reproducible Science (CRS) & Epidemiology, Biostatistics and Prevention Institute (EBPI), University of Zurich, Zurich, Switzerland; 5 Nuffield Department of Population Health, University of Oxford, Big Data Institute, Li Ka Shing Centre for Health Information and Discovery, Oxford, United Kingdom; 6 KG Jebsen Centre for Neurodevelopmental Disorders, University of Oslo, Oslo, Norway; 7 Department of Psychiatry and Psychotherapy, University of Tübingen, Tübingen, Germany; Museo Storico della Fisica e Centro Studi e Ricerche Enrico Fermi, ITALY

## Abstract

Mental disorders often emerge during adolescence and have been associated with age-related differences in connection strengths of brain networks (static functional connectivity), manifesting in non-typical trajectories of brain development. However, little is known about the direction of information flow (directed functional connectivity) in this period of functional brain progression. We employed dynamic graphical models (DGM) to estimate directed functional connectivity from resting state functional magnetic resonance imaging data on 1143 participants, aged 6 to 17 years from the healthy brain network (HBN) sample. We tested for effects of age, sex, cognitive abilities and psychopathology on estimates of direction flow. Across participants, we show a pattern of reciprocal information flow between visual-medial and visual-lateral connections, in line with findings in adults. Investigating directed connectivity patterns between networks, we observed a positive association for age and direction flow from the cerebellar to the auditory network, and for the auditory to the sensorimotor network. Further, higher cognitive abilities were linked to lower information flow from the visual occipital to the default mode network. Additionally, examining the degree networks overall send and receive information to each other, we identified age-related effects implicating the right frontoparietal and sensorimotor network. However, we did not find any associations with psychopathology. Our results suggest that the directed functional connectivity of large-scale resting-state brain networks is sensitive to age and cognition during adolescence, warranting further studies that may explore directed relationships at rest and trajectories in more fine-grained network parcellations and in different populations.

## Introduction

The brain undergoes tremendous changes throughout life, where childhood and adolescence is a particularly sensitive developmental period for brain maturation processes [1]. A vital part of the brain’s maturation happens in the functional networks that show pronounced reorganization in order to facilitate neural efficiency and integration of information, as indicated by functional magnetic resonance imaging (fMRI) studies in healthy children and young individuals as well as in clinical populations in the same age range [2–6]. This supports that this time period is a sensitive phase, where there are large alterations in the functional interconnections between brain regions, denoting the functional connectome, that link to when different mental disorders emerge [2].

The primary method to study the functional connectome so far has been to estimate static functional connectivity between brain regions using measures such as correlation, which evaluates undirected link strengths using stationary coefficients with the assumption that the signal is stable during the scan duration. Using this approach, research has shown that the functional interconnections in healthy children and adolescents become more specialized through pruning of connections [7]. It has also been shown that resting-state networks (RSNs) also become more integrated and distinct during this time-period [8], and evidence has indicated aberrant connectivity in individuals with pre-clinical psychiatric symptoms, with a delay in connectome maturation and distinctiveness [9]. This is in line with the brain dysconnectivity hypothesis, which posits a breakdown in the brain’s functional integration in mental disorders [10–13].

Recently, estimating connectivity direction [14] has received interest as a field, as it could add insight into the connections between brain regions that we know are directed [15]. Directed functional connectivity (dFC) yields information about topology and how neural information is passed between different brain regions by estimating in which direction the neural activity is transferred between networks and has been shown to explain variance beyond static functional connectivity measures [16].

For developmental research, Stevens, Pearlson [17] investigated directed connectivity using resting-state fMRI data from healthy adolescent and adult participants, and found the proportion of significant directed relationships between network components to vary with age, identified for instance for edges between the default mode and prefrontal-parietal regions. Further, Zhou, Friston [18] examined three networks and directed relationships at rest in youths and young adults, and found that the default mode network’s activity is inhibited by the salience network and the dorsal attention network, whereas the default mode network stimulates both the salience network and the dorsal attention network. Additionally, a study using correlation-purged Granger causality that examined 26 healthy young participants, discovered that the default mode network contained major hubs, the anterior prefrontal cortex was a major recipient of input alongside the hippocampus, and that there are significant outputs from the anterior insula and the middle temporal region directed at the anterior prefrontal cortex [19]. Further, by use of task-fMRI, Riley, Chen [20] examined connectivity direction in a neurodevelopmental sample, where they observed differences between sexes in relation to how memories are encoded in the hippocampus, in line with general findings of maturation trajectories for boys and girls in this developmental period [21–23]. In another neurodevelopmental study by Hwang, Velanova [24], the authors uncovered that improvements in inhibitory control were linked to strengthening of top-down connectivity for regions implicated in cognitive control networks, while similar findings were reported in relation to top-down processing for a language network [25], highlighting the importance of examining associations in the context of age-dependent effects. Further, it has been shown that there are differences in directionality in adolescent boys with externalizing behavior disorders in comparison with controls [26]. Likewise, alterations in connectivity direction in resting-state fMRI (rsfMRI) data have been observed for clinical samples, where children with autism and Attention Deficit Hyperactivity Disorder (ADHD) show a common pattern implicating Broca’s area across clinical groups, along with distinctive patterns of functional connectivity specific for given disorders [27]. In addition, others have found higher directed connectivity from left to right superior parietal lobule to be associated with an improved shift and emotional control across healthy children and clinical participants [28].

However, the literature on connection directionality is scarce, and selected methods utilized for estimation of dFC has been shown to be biased and prone to yield spurious findings as it is influenced by differences in regional variability in the hemodynamic response [29,30], calling for validation of earlier results and for new insights using novel approaches. In particular, we do not currently know how and to what extent the direction flow in the communication between brain networks is implicated in mental traits and disorders. Given that cognitive deficits are seen across mental disorders, connecting these behavioral and clinical characteristics is crucial for gaining a better understanding of how higher-order cognitive functioning is characterized in children and adolescence. Such integrative understanding is also important given that no consensus has been reached when it comes to the disorder-specific connectivity alterations that characterize particular mental disorders and the known overlap of symptoms [31,32], and genetic pleiotropy between disorders with shared genetic etiology [33,34]. Taken together, these findings show the importance of using continuous symptom characteristics to map brain fMRI connections to clinical profiles, in line with The Research Domain Criteria project (RDoC), launched by NIMH to address the need for a new strategy for defining mental disorders [35].

To examine phenotypic relationships and connectivity directionality, as they relate to brain development and dimensional psychiatric measures, we made use of Dynamic graphical models (DGM). DGM is a Bayesian approach for examining dFC using dynamic linear models (DLM) [36]. As such, it implements a state space model that is linear and Gaussian in form. This includes statistically stationary properties as it uses a hidden Markov modelling approach, although it incorporates time varying coefficients and as a result can provide information about directionality in the form of a binary view of coupling between two brain regions for each connectivity direction. As such. the outcome indicates if there is an influence or not between two networks. DGM differs from the conventional idea of Granger causality, which has been the most widely applied approach in this field so far, in that it takes into consideration instantaneous interactions. To establish face validity, Schwab, Harbord [36], who first proposed the DGM method, assessed the consistency and reliability of DGM networks in a sizable sample of HCP rsfMRI data in addition to simulating systematic lags in the hemodynamic response in various brain regions to ascertain how these lags would affect dFC estimates. They observed that DGM worked well in these network simulations.

In a previous study we used this method to replicate and extend analysis by examining dFC from more than 11000 adults [37]. We confirmed prior results of a two-way directed connection between the visual medial and lateral networks as well as information flow from many large-scale brain networks into the cerebellum. Additionally, there was large-scale associations between dFC and sex as well as age, with the sensorimotor network showing the highest effects of age. In addition, visual, auditory, and sensorimotor nodes were linked to mental health in UK Biobank participants.

Here, we used DGM and publicly available rsfMRI data from the Healthy Brain Network (HBN) project [38] to study brain network information flow in neurotypical and non-typical children and adolescents and its associations with age, sex, cognitive abilities, and psychopathology. We hypothesized that dFC between networks would have a positive association with age [24,25], that there would be differences between females and males in the maturation of brain networks and its information flow [20], as well as alterations associated with information flow for control networks as these nodes are central to a range of disorders [39–44].

## Methods

### Study samples

The HBN is a project organized by the Child Mind Institute [38] and is a resource targeting novel insight into the critical time period when psychiatric and mental disorders emerge. The HBN consortium aims to include 10,000 individuals in the age range of 5–21 years from the New York area, where participants are included by use of announcements that are distributed to community members, educators, local care providers with the addition of sending information via email lists and events to parents, encouraging participation of children with clinical concerns to this study [38]. Data on these individuals include a package consisting of MRI scanning, genetics, electroencephalography (EEG), eye-tracking, as well as biological testing and a neuropsychological battery consisting of cognitive, lifestyle indices, behavioral and psychiatric domains in addition to actigraphy and voice and video interviews [38]. Exclusion criteria include serious neurological disorders, neurodegenerative disorders, acute encephalopathy, hearing or visual impairment, lifetime substance abuse that necessitated chemical replacement therapy/acute intoxication at time of study, recent diagnosis of a severe mental disorder or manic/psychotic episode within the last 6 months without ongoing treatment, in addition to the onset of suicidality/homicidality where there is no current, ongoing treatment [38]. All participants over the age of 18 years provided signed informed consent, while legal guardians signed informed consent for participants under the age of 18, in addition to participants giving a written assent [38]. The Chesapeake Institutional Review Board approved the study (https://www.chesapeakeirb.com/).

### MRI acquisition and preprocessing

MR data was collected by the study team of HBN, where we included MRI data from the following sites: Rutgers University Brain Imaging Center (RUBIC), Citigroup Biomedical Imaging Center (CBIC) and a mobile scanner located in Staten Island. MRI data was collected using one scanner at each site, giving a total of 3 scanners comprising our sample. Rutgers applied a Siemens 3T Tim Trio scanner, while CBIC utilized a Siemens 3T Prisma, and both sites applied the same MRI parameters, where resting- state blood-oxygen-level-dependent (BOLD) fMRI data was collected for each subject using a T2*-weighted BOLD echo-planar imaging (EPI) sequence with a repetition time (TR) of 800ms, echo time (TE) of 30ms, multiband acceleration factor = 6, 60 number of slices with the rsfMRI session consisting of 375 volumes and with voxel size = 2.4×2.4×2.4 mm. The third mobile scanner located in Staten Island used a 1.5T Siemens Avanto system equipped with 45 mT/m gradients [38], where the following parameters were implemented; TR = 1.45s, TE = 40ms, multiband acceleration factor = 3, number of volumes = 420, slices = 54, resolution in mm = 2.5×2.5×2.5 mm (for more information about the MRI parameters, see http://fcon_1000.projects.nitrc.org/indi/cmi_healthy_brain_network/MRI%20Protocol.html). Raw imaging data was downloaded from the HBN database (http://fcon_1000.projects.nitrc.org/indi/cmi_healthy_brain_network/sharing_neuro.html), and analyzed on the secure data storage and computing facilities (TSD, https://www.uio.no/tjenester/it/forskning/sensitiv) at University of Oslo. FSL 6.0.3 image processing tools (http://fsl.fmrib.ox.ac.uk), outlined in detail below, were used for the functional data while T1-weighted data, which was applied as an intermediate in the registration, was processed using FreeSurfer 5.3 (http://freesurfer.net), including removal of non-brain tissue.

As part of the HBN MRI protocol, multiple T1-weighted sequences were acquired for each subject, and we used MRIQC version 0.14.2 [45] for automated quality assessment (N = 2427, The CUNY Advanced Science Research Center scanning site was included for the MRIQC stage, yet this site was dropped at a later stage due the low sample size, N = 22). MRIQC estimation is gleaned from 14 image- quality metrices that are based on noise measurements, information theory, measures targeting specific artifacts as well as other image estimates such as the full-width half maximum for deriving image blurriness [45]. From this, T1-weighted images with the best image quality metrics, based on the classifier ratings, were used as input for registration, while structural scans that were flagged as low quality (classifier rating score >0.5, N = 160) were manually checked and excluded (N = 117). In addition, 22 subjects had errors when they were run through FreeSurfer and after manually inspecting these datasets, these were also omitted due to motion artefacts and/or having MRI findings yielding errors to the automated segmentation performed as part of the FreeSurfer pipeline. We also made a global mask of the T1 data where we manually checked subjects that had low coverage to confirm that regions of interest for each subject was within the global mask, excluding another 95 subjects leaving a total of 2171 datasets that had a usable T1-weighted sequence for registration.

Two of the MRI sites, RUBIC and CBIC, had two resting-state scans acquired as part of the same MRI session. As the DGM method benefits from more time points, we merged the time series for subjects with two resting state scans together to leverage all available resting-state data. This was done prior to implementing FEAT as a means of optimizing the data by improving the spatial alignment between the sessions, and to have the full set of volumes to inform FIX. Furthermore, the first five volumes for the fMRI dataset were discarded. We preprocessed fMRI data using FSL, including motion correction and brain extraction. FSL’s FEAT [46] included spatial smoothing with a Gaussian kernel FWHM of 6 mm and a high-pass filter cutoff of 100. FMRIB’s Nonlinear Image Registration tool (FNIRT) was used to register fMRI volumes to standard space (MNI-152) with the T1-weighted volumes as intermediates.

We also implemented a cleaning step for fMRI data, where we made a global mask for the fMRI datasets (which indicated data coverage across participants), and from this we excluded 128 of 1813 datasets with poor coverage. To reduce the influence of noise in the data and increase the temporal Signal-to-Noise Ratio (tSNR) [9], we removed artefacts by use of ICA-AROMA, a classifier that identifies and removes motion specific noise in fMRI data [47,48]. Afterwards, ICA was rerun and FSL’s FIX (FMRIB’s ICA-based X-noisiefier [49,50]), was used with the recommended threshold of 20 to remove remaining motion confounds and other artefacts in the data. Further, data was temporally demeaned and variance normalized [51], and the quality controlled fMRI dataset (N = 1685) was submitted to a group ICA, utilizing FSL’s Multivariate Exploratory Linear Optimized Decomposition into Independent Components (MELODIC) tool [51,52], where 25 components were extracted from the ICA and used for further analysis. Dual regression was applied to estimate individual spatial maps and corresponding time series from the group ICA [51,53], which were used as input for the DGM analysis.

### Measures of mental health and cognition

From the HBN data release, N = 1685 participants were included after quality assessment. Out of these, information on age at MR was missing for N = 45, sex for N = 9, and cognitive/clinical information for 496 individuals. The participants were 5–22 years (mean: 11.5, years, sd: 3.51 years) and 37.9% were females. This data was used to study group-level patterns of dFC (average connectivity matrix). For the subsequent associations with age, sex, cognition and mental health, we restricted the analysis to a subset based on data availability. Thus, the final sample for the association analyses comprised N = 1143, individuals aged 6–17 years (mean: 10.7 years, sd: 2.62 years, 37.4% females, where N = 83 where from the Staten Island site, N = 503 from CBIC and N = 557 from RUBIC scanning site (see supplementary material; S2 Fig for age distributions within scanner sites). A large proportion of the sample had a diagnosis (N = 1012), while N = 105 did not have a diagnosis, N = 23 dropped out before a diagnosis could be determined, and N = 3 was missing information for clinical consensus diagnosis data and had as such not received a diagnosis, see supplementary material; S1 Fig for further details.

**Fig 1:**
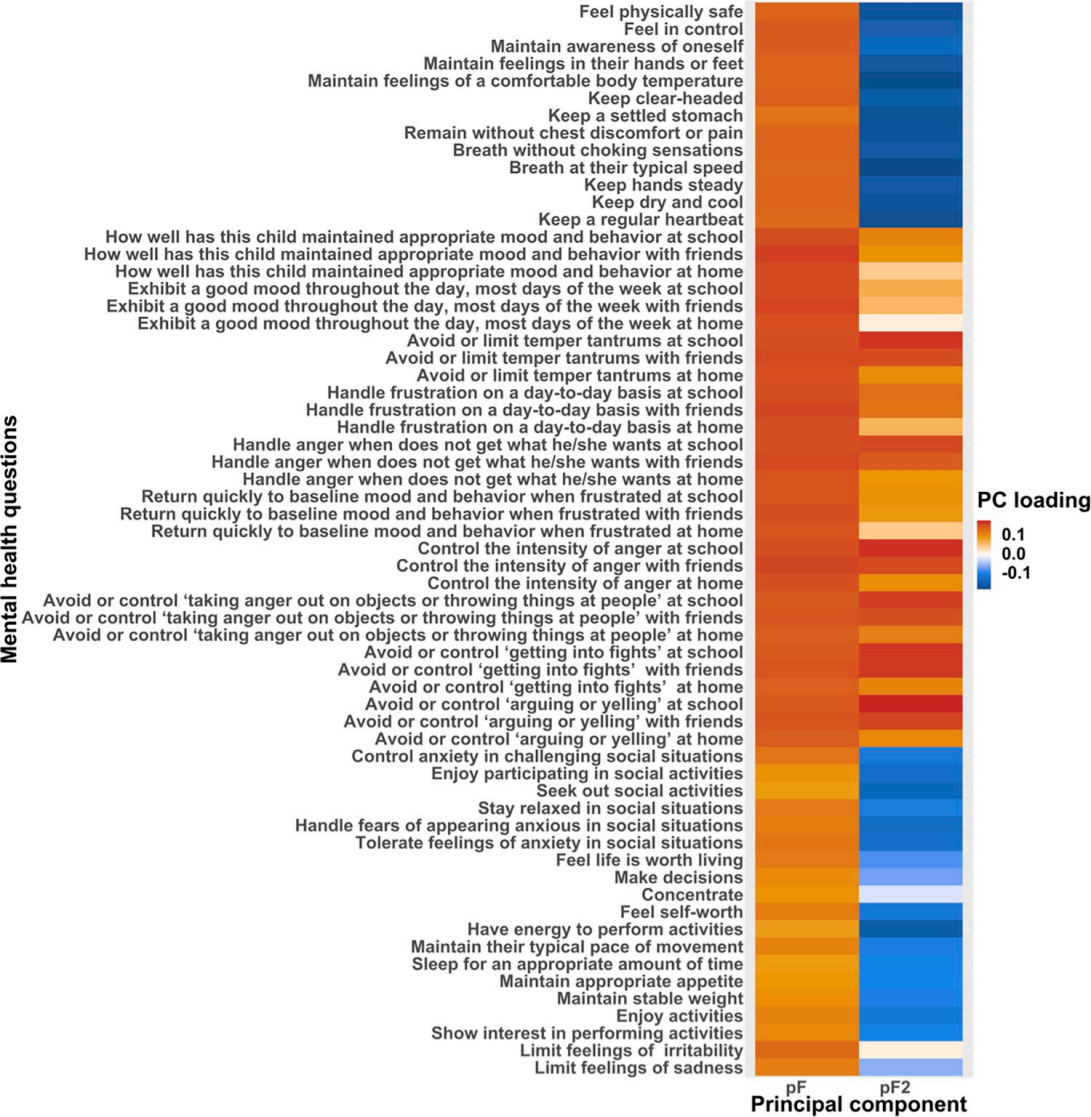
Principal component analysis performed on the E-SWAN questionnaire taken as part of the HBN protocol. For visualization purposes, only the components included in our analysis are shown. We used the first two principal components as proxies of psychopathology, referred to as “pF” and “pF_2_”.

We used the full-scale intelligence quotient (FSIQ) from the Wechsler Intelligence Scale for Children (WISC-V) taken for participants aged 6–17 years as a proxy for cognitive ability. This composite score includes the following domains; visual spatial, verbal comprehension, fluid reasoning, working memory, and processing speed [54]. Mental health was measured on a continuum including both healthy subjects and patients as it is difficult to uncover robust findings for psychiatric diagnosis that constitutes heterogeneous disorders, showing a wide range in symptoms, severity, duration and prognosis, and as patients often have more than one diagnosis. Such heterogeneity is also reflected in the brain, making the search for biological markers a complex task. As such, for mental health, we performed a principal component analysis (PCA) on The Extended Strengths and Weaknesses Assessment of Normal Behavior (E‐SWAN), which has been shown to be a valid psychometric assessment tool for investigating behavior underlying DSM disorders [55]. E-SWAN domains include depression, social anxiety, disruptive mood dysregulation disorder (DMDD), and panic disorder. We excluded 3 of the items relating to panic disorder from the questionnaire that had a high degree (90%) of missing values, giving a total of 62 items for the PCA analysis. The remaining items had available data for 2626 participants with no missing values. We performed PCA using the “prcomp” function in R, where the first PC, often denoted as the p-factor or pF [56], explained 43.6% of the variance (Fig 1). From the loadings from the PCA, this component was associated with items related to self-control and depression/anxiety (Fig 1). In accordance with other studies showing more than one factor being of importance [57,58], we also included the second principal component referred to as pF_2,_ which was associated with items relating to mood dysregulation. This component explained 11.3% of the variance.

### Network analysis

**Fig 2:**
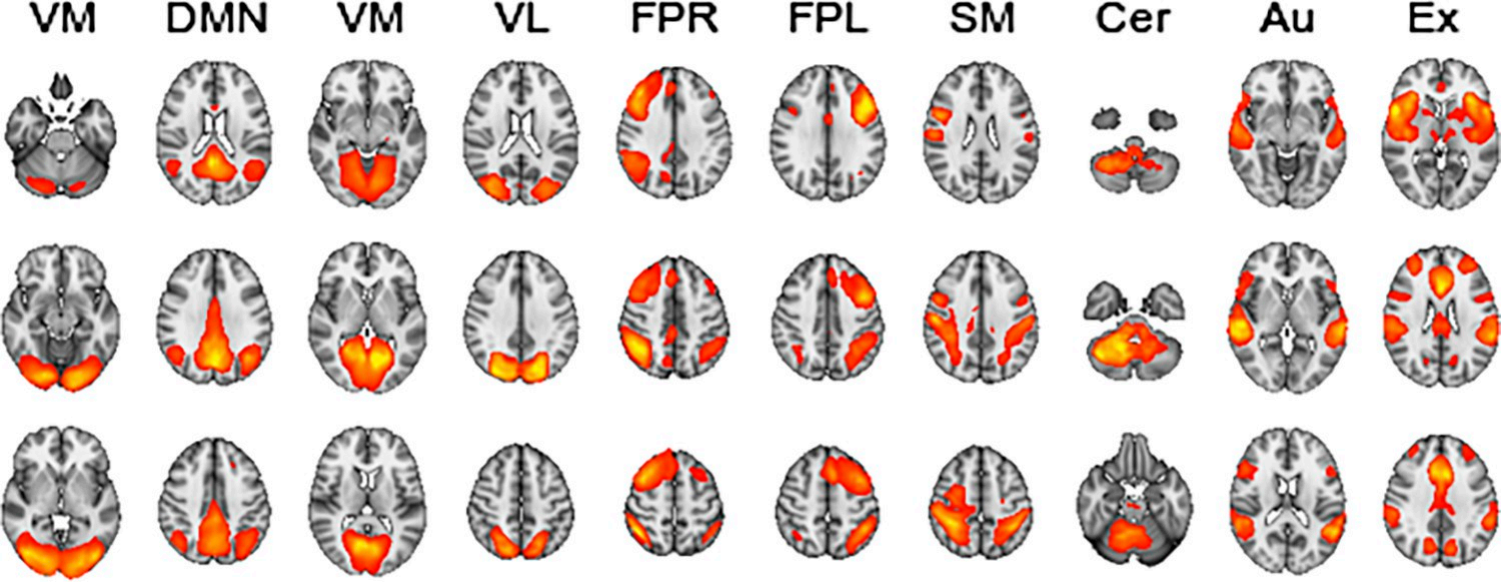
Group-level maps for the 10 selected independent components included in the analysis. The z-score maps threshold is set at 4. VO, visual occipital; DMN, default mode; VM, visual medial; VL, visual lateral; FPR, frontoparietal right; FPL, frontoparietal left; SM, sensorimotor; Cer, cerebellum; Au, auditory; Ex, executive control network.

We chose ten resting-state networks from the model order of 25 for inclusion in the dFC analysis. These networks were chosen based on spatial correlation and manually ensuring overlapping ICs with the ten RSNs reported by Smith, Fox [59]. The ten networks comprised the default mode network, cerebellar, visual occipital, visual medial, visual lateral, right frontoparietal, left frontoparietal, sensorimotor, auditory, and the executive control network (Fig 2). These networks were also used in previous DGM studies [36,37]. Including the same nodes, referring to the independent components included, and utilizing the same model order, allowed us to compare results with prior findings. It also permitted us to integrate the current results obtained from a childhood and adolescent sample with previous adult studies. The included timeseries for each node were mean centered before estimating dFC from individual level RSN time series using the DGM package v1.7.2 in R. DGM is a set of regressions with time-varying coefficients (DLMs) for every receiving node. The receiver node refers to the network that receives information from a sending node that transmits information between a node pair. For each DLM, all possible models for the sender nodes are tested and the model with the highest model evidence is selected. Consequently, a model is stated by the sending networks of a node, giving a binary outcome. DGM has uncovered meaningful trajectories in rsfMRI mice data where DGM revealed a link between areas in the hippocampus that fed information to the cingulate cortex, in line with previous studies using viral tracers to delineate directed anatomical connectivity in mice [36]. Additionally, even with systematic hemodynamic lag confounds being introduced in the data, network simulations demonstrated a sensitivity of 72%–77% for the DGM approach [36].

### Statistical analysis

We included all available rsfMRI scans for the group ICA, as a higher number of subjects is beneficial for yielding more robust ICs, while restricting the association analyses to the subset that had all covariates available. We performed the same analysis as previously reported in an adult sample [37], examining dFC on the edge- and node-level. Edge-level analysis deployed logistic regression for every connection of the directed network using directed connectivity as the response variable and testing for associations with age, age-orthogonalized age squared (age^2^, using the poly function in R), sex, cognitive abilities, mental health, tSNR, motion and scanning site (as data was acquired at multiple scanners). These covariates were included into one model (see supplementary material; S5 Fig, for additional analyses examining potential multicollinearity for covariates included in the model). P-values were Bonferroni corrected for a number of 90 analyses on the edge-level (Bonferroni level P < .00056, unique connections in a 10x10 network)).

In addition, we performed node-level analysis to examine which networks overall send and receive information to each other [37]. We calculated the number of output connections or outgoing edges (referred to as out-degree), with edges denoting the directed connection between networks at the subject level, and the number of input connections or incoming edges (referred to as in-degree) for a given node. We performed linear regression using in-degree and out-degree as dependent variables and the same independent variables as used in the logistic regression on edge-level. P-values were Bonferroni corrected for a number of 10 analysis on the node-level (Bonferroni level P < .005).

## Results

**Fig 3:**
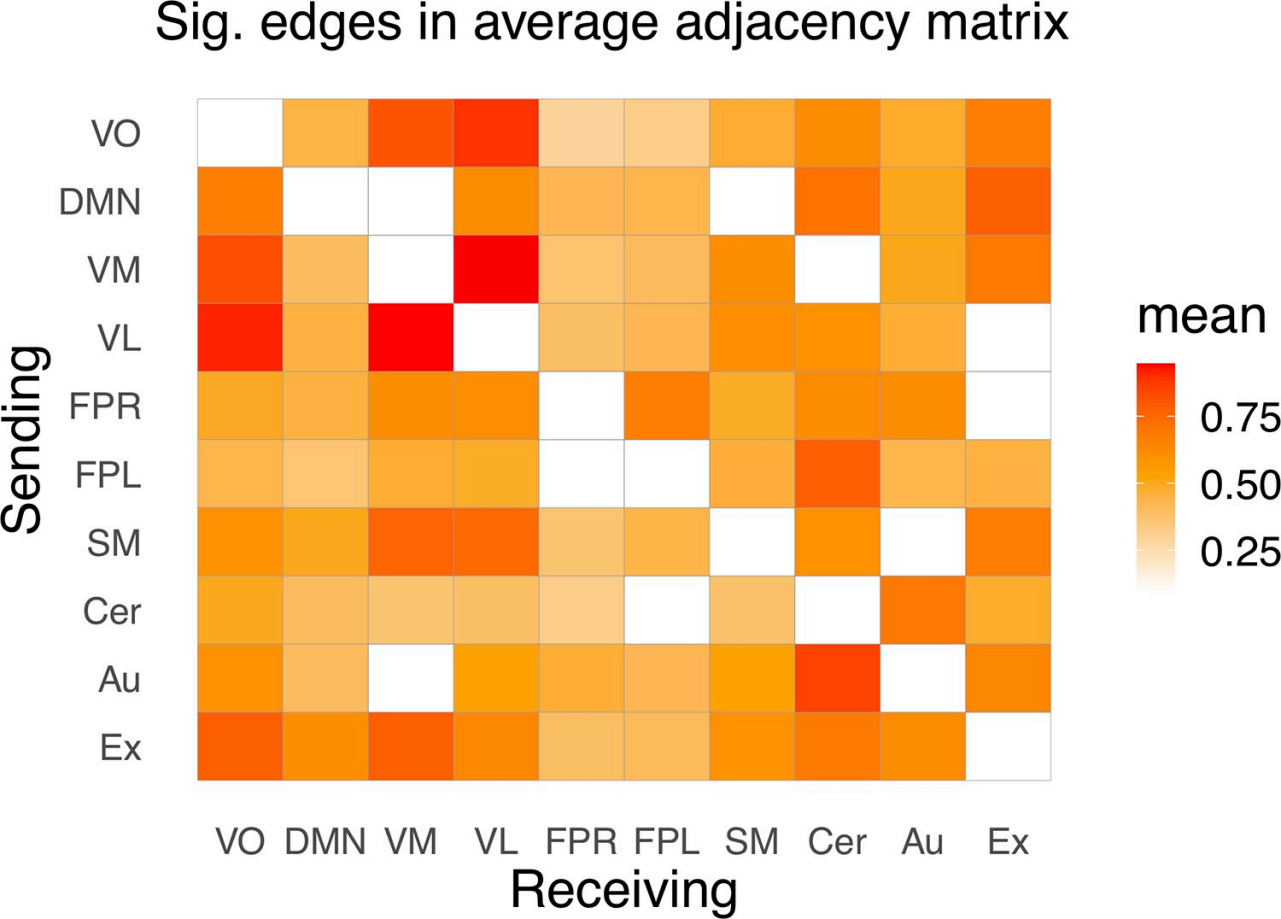
Average directed connectivity for HBN individuals with the matrix displaying the significant percentages of edges between the 10 RSNs included for analysis. VO, visual occipital; DMN, default mode; VM, visual medial; VL, visual lateral; FPR, frontoparietal right; FPL, frontoparietal left; SM, sensorimotor; Cer, cerebellum; Au, auditory; Ex, executive control network. The y-axis indicates the sender node, and the x-axis indicates the receiver nodes. Significance was assessed using a binomial test as implemented in the binom.nettest function in R, with a predefined FDR threshold of 5% and the hypothesized probability p0 = .56.

Fig 3 depicts the average directed functional connectivity matrix across all individuals. Several connections indicated bi-directionality in dFC, especially directionality estimates for the visual lateral and visual medial networks where directed connectivity from the visual medial to the visual lateral network was present in 94.4% of individuals, while connection from the visual lateral to the visual medial was present in 94.2%. In addition, there was overall a high number of participants that showed a reciprocal relationship between the visual occipital to the visual medial and the visual lateral regions (visual occipital to visual lateral: 89.1%, visual occipital to visual medial: 80.2%) and opposite (visual lateral to visual occipital: 91.5%, visual medial to visual occipital: 82.7%). These bi-directional relationships are in alignment with patterns of dFC found in adults from the UK Biobank sample [37]. However, we did not observe the bi-directional dFC found previously for adults between the default mode network and the right frontoparietal network. Further, when observing receiver networks, the left frontoparietal showed the same as found for adults, that it did not receive information to a large extent [37], coherent with our findings in youths. Additionally, the edge most present across all individuals, aside from the visual networks, was the cerebellar network which receives input from the auditory network (85.6%). This connection was also relatively strongly expressed in the other direction (68.6%). Moreover, the left frontoparietal sent information to the cerebellar network, and this edge was present in 77.7% of individuals. Yet, in this neurodevelopmental sample we did not observe that the cerebellar and auditory network overall were mostly receivers, as found for adults [37].

### Effects of age and cognition on dFC measures

**Fig 4:**
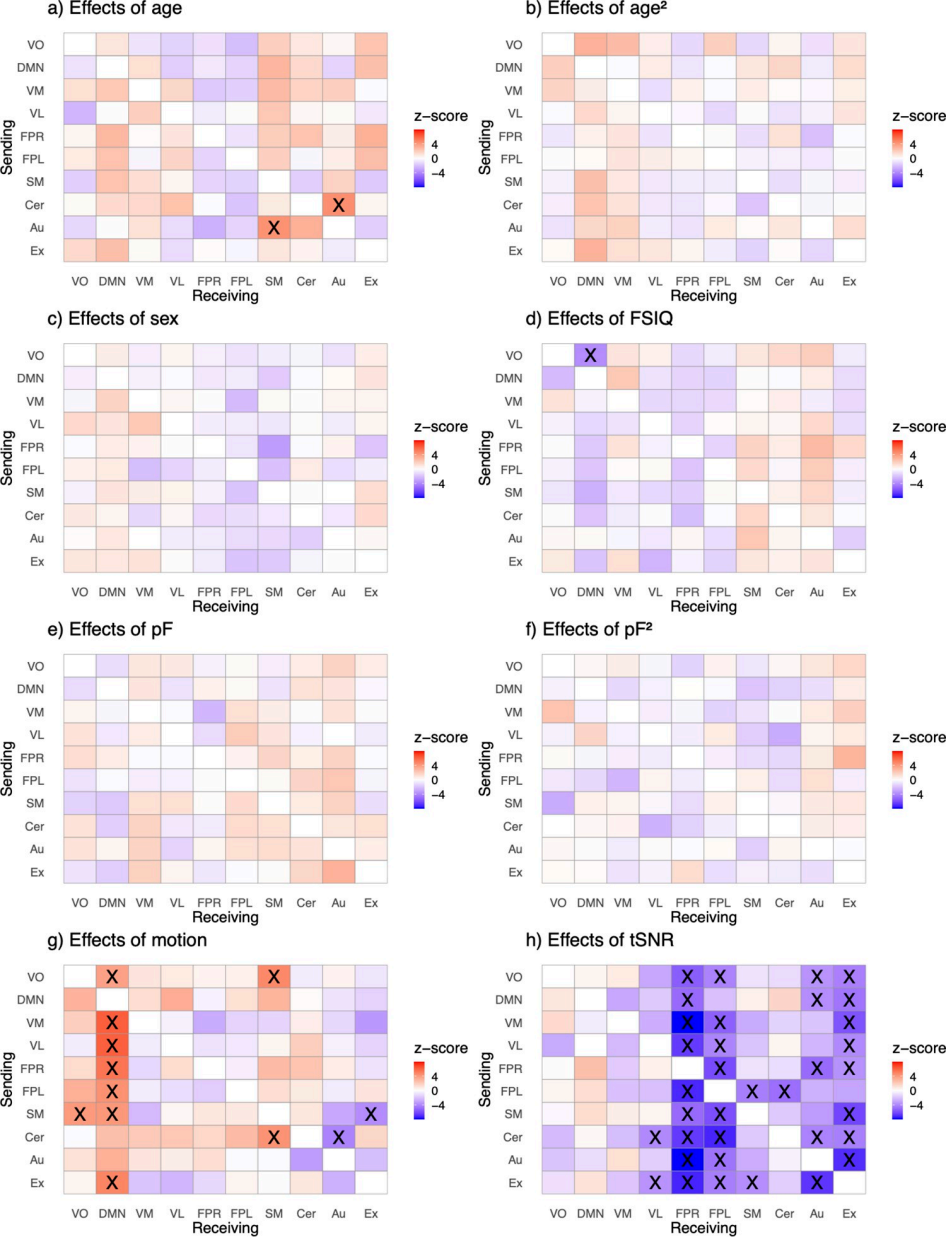
Matrices showing the effects of age (a), age^2^ (b), sex (c), intellectual abilities (FSIQ) (d), mental health (pF and pF_2_) (e,f), motion (g), and tSNR (h) on directed connectivity. The analysis was performed in HBN data that had no missing values (N = 1143, 6–17 years, df = 1132). Significant edges following Bonferroni correction are marked as X. The legend shows the 10 RSNs included in the analysis; VO, visual occipital; DMN, default mode; VM, visual medial; VL, visual lateral; FPR, frontoparietal right; FPL, frontoparietal left; SM, sensorimotor; Cer, cerebellum; Au, auditory; Ex, executive control network. The y-axis indicates the sender node, while the x-axis refers to the receiving node. The colors reflect the z-value for the corresponding effects where red indicates a positive association and blue a negative association.

Edge-level analysis of dFC showed significant effects of age, in line with our hypothesis of age being associated with the functional networks’ maturation processes occurring in childhood and adolescence (Fig 4; S1–S6 Tables show z-scores and p- values, and S3 and S4 Figs show effects of scanner, while S6 Fig show results after false discovery rate (FDR) correction). Specifically, we observed a positive association with age and dFC from the cerebellar network to the auditory network. Correspondingly, the auditory network sends more information with higher age to the sensorimotor node. For cognitive test performance and dFC, we observed a significant negative association between the visual occipital and default mode network, with the default mode network receiving less information from the visual occipital network with higher cognitive test performance.

**Fig 5:**
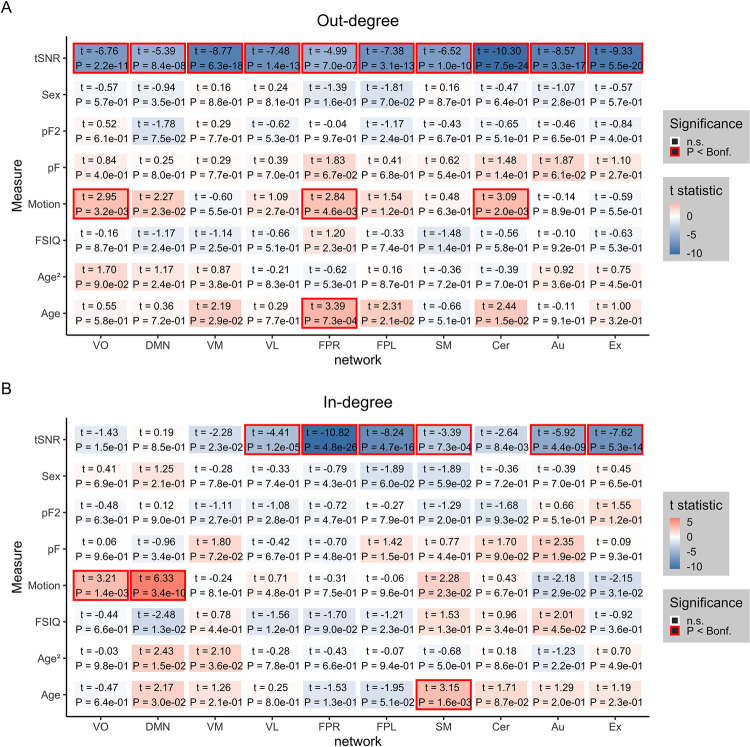
Associations on the node level (N = 1143, 6–17 years, df = 1132). a) Out-degree matrix with corresponding effects of covariates age, age^2^, sex, cognition (FSIQ), mental health (pF and pF_2_), tSNR, and motion in HBN data. b) In-degree matrix with corresponding effects for the same covariates as in panel a). The colors reflect the t-value for the corresponding effect where numbers inside the boxes indicate t-statistic and p-value, and significant effects are marked with a red border following Bonferroni correction (p < 0.05).

On the node-level, we assessed similar phenotypic associations for the out-degree and in-degree of the networks. Confirming edge-level results, age was significantly associated with the in-degree (number of input connections) of the sensorimotor network (Fig 5B), indicating that the sensorimotor network overall receives more information with increasing age. Additionally, for out-degree (number of output connections) there was a significant positive association for age and the right frontoparietal network, signifying that the right frontoparietal network overall sends more information with increasing age. As expected, there was significant effects for the control variables scanning site (S3 and S4 Figs), tSNR and motion on edge and node-level.

Based on prior studies, we expected that mental health would be associated with dFC for control networks, however, this was not observed. We also expected sex-related differences in the maturation of brain networks, but this was not found.

## Discussion

As it is unknown how dFC between large-scale intrinsic networks evolves in the maturing brain and as part of psychopathology, we aimed to broaden previous research on dFC to a sample of typical and non-typical youths and examine connectivity directionality and associations with age, sex, cognition, and mental health in a crucial developmental period. Our analysis revealed marked dFC patterns for the visual networks. Specifically, we observed a reciprocal dFC between the visual medial and visual lateral networks in the investigated sample of children and adolescence. This is in alignment with previous findings in adults [36,37], and coherent with primary sensory and motor cortical regions maturing first, and being present early on in children [60]. In contrast, we did not find that the cerebellum or auditory networks are mostly receivers of information as observed in adults [36,37]. However, apart from the visual circuits, the cerebellar network received most information overall from the other transmitter networks, potentially indicating its role as a receiver, as found for adults.

We observed significant associations between age and directed connectivity both at edge and node-level. At the edge-level and with increasing age, the cerebellar node had higher dFC to the auditory network, while the auditory network sent more information to the sensorimotor network. Of note, these connections demonstrating age-dependent dFC have been implicated in neurodevelopmental disorders such as autism, which is characterized by symptoms related to hyper- and hypo sensitivity in relation to sensory modalities [61]. Moreover, it has been reported that the cerebellar network is mostly a receiver of information from other attention and memory related regions in children with ADHD when estimating directed connectivity in rsfMRI using a seed-based approach [62]. The average connectivity across participants indicated that the left frontoparietal network sent information to the cerebellar network in 77.7% of the participants, which is in line with the aforementioned findings. Hence, this could be an interesting avenue to investigate in relation to potential biomarkers in future studies.

Likewise, on the node-level, the sensorimotor network received more information with increasing age, while the right frontoparietal network sent more information with higher age to the other networks overall. These results indicate that directionality estimates in both bottom-up and top-down processing are involved in the transitional phase from child to adulthood across patients and healthy individuals. This is in line with prior findings implicating these networks in normal functional development [63]. Similarly, alterations in the right frontoparietal network have been associated with ADHD, where children and adolescent ADHD combined type patients show a reduction in right frontoparietal regions, when probing spatial working memory by use of task-fMRI [64,65].

For cognitive abilities and dFC, we observed a significant negative association between the visual occipital and default mode network, with the default mode network receiving less information from the visual occipital network with higher cognitive test performance. This could signify that individuals that were more focused on the task at hand benefited from less directed connectivity flowing from visual occipital to the default mode network, potentially facilitating effective whole-brain connectivity which has been linked to cognitive efficiency [66]. However, it is challenging to delineate dFC as this could be disrupted for many connections and give rise to different alterations in connectivity. Nonetheless we did expect to observe associations for control networks in relation to cognitive abilities as this has been reported by others [67–69]. Interestingly, the core executive function cognitive flexibility, has in healthy adults been associated with strong within-system connectivity in higher order systems and from these networks to primary-sensory motor systems when estimating dFC [16]. Altered within network connectivity in relation to cognitive flexibility and in general has also been reported for neurodevelopmental disorders [27,28]. Here, we estimated dFC between networks with different functions rather than within-system connectivity (with the exception of the visual networks). In addition, we used a normed cognitive composite test score as a proxy for cognition. However, this may not be an optimal approach as test norms could have limitations when it comes to representativeness and number of subjects included in the normative sample, making it less sensitive for clinical populations and for the lowest scoring percentile.

Further, we utilized two general p factors for measuring symptoms in this sample. These psychopathology loadings were related to self-control and depression/anxiety (pF) and items pertaining to mood dysregulation (pF_2_). Top-down control functions for cortico-limbic regions have been linked to modifications for symptoms relating to mood dysregulation in development. For instance, it has been shown that increased centromedial amygdala-rostral anterior cingulate cortex functional connectivity is related to a higher level of anxiety and depression symptoms during early adulthood, while increased structural connectivity in centromedial amygdala-anterior ventromedial prefrontal cortex white matter was linked to augmented symptoms of anxiety and depression during late childhood [70]. We did not observe significant associations between dFC and symptom burden for any of the networks. Our dimensional approach of estimating PCA on symptom data represents both healthy subjects and individuals with a psychiatric disorder on a symptom continuum rather than as cases and control. Such a dimensional approach may better capture intermediate phenotypes which map to brain biology and the neuronal mechanisms underlying the symptoms, compared to diagnostic categories [71,72]. While the lack of significant associations in our study indicates that between-network dFC in large-scale brain networks is not sensitive to mental health in this sample, future research will need to investigate dFC both within- and between nodes and as part of distinct subnetworks, and by different symptom measures, in addition to also considering structural connectivity.

When controlling for confounds, we observed widespread effects of motion and tSNR on dFC, as expected for the here studied age group. We took meticulous measures to clean the data prior to dFC modelling, and we performed additional supplemental analyses in which we reran the main analysis after excluding those individuals with greatest mean relative displacement (motion) or low signal to noise ratio. S7 and S8 Figs indicates similar results of these analyses compared to the main analysis.

We also expected to find differences between sexes in relation to directed connectivity as this has been found previously in adolescence [20] using task-fMRI, and when examining dFC in adults [37]. However, we did not find any significant sex differences in dFC on edge- or node-level in rsfMRI. This may partly relate to sample characteristics such as the uneven sex distribution and the inclusion of patients in our sample.

## Limitations

As with any MRI study, participant motion during scanning may confound the results, especially as it is known that both young individuals and those with a psychiatric diagnosis on average move more [73]. We therefore implemented a stringent correction pipeline, where we implemented several steps of ICA and machine learning based cleaning (FIX and AROMA). We also included the mean relative motion measure from FSL as a covariate in our analysis step. Another relevant confound effect that could potentially bias our results and the comparison to previous work is variability in the ICA decompositions. We performed ICA in the study sample and selected components based on similarity to components reported by Smith, Fox [59]. While this approach yielded overall good comparison to previous work, subtle differences between decompositions may retain and might impact the derived dFC patterns. For instance, sensorimotor nodes were divided into three independent components where the node with the best spatial overlap with the sensorimotor component in Smith, Fox [59] was chosen. Most likely including another sensorimotor related node would change the results of our analysis. Hence, our results call for follow-up analysis in more fine-grained networks. In this framework, different ways to parcellate the brain could be tested.

The majority of the individuals in the sample were diagnosed with a mental disorder. This was beneficial when examining associations of psychopathology on directionality estimates as it yielded a high number of patients in each group, but makes it difficult to delineate if average patterns of information flow are for typical or non-typical functional development. Some of our findings are not consistent with prior studies targeting static brain connectivity in youths, such as for example the joint reorganization of default mode network and frontal brain regions [74]. To what degree these inconsistencies relate to methodological differences or reflect non-typical development remains to be investigated. Stevens, Pearlson [17] examined neurotypical trajectories in adolescents and young adults and reported a general pattern of decrease in Granger causality relationships between RSNs in association with age. However, concerns have been raised about using Granger causality estimates for fMRI data, yielding an unclear picture of dFC findings in healthy development.

In addition, medication may have had an effect on our results, as participants were asked to discontinue stimulant medication but were still enrolled if they chose not to discontinue due to personal reasons or if recommended by their physician.

Also, DGM is an observational approach and we can not infer the exact signal that is transferred between nodes. Hence, it could for instance be that we are measuring an inhibitory signal or a mix of signal processing occurring under an unconstrained condition such as rsfMRI. On a methodological note, DGM reflects instantaneous relationships and does not only look at lagged relationships, however sensitivity of DGM drops depending on the total offset between a node pair and can as such influence the estimation of these interactions. Further, DGM estimates binary connections, which may have reduced the sensitivity of the association analyses. Lastly, whether differences in findings compared to previous studies in adults are of methodological origin, such as differences in preprocessing pipelines or network definitions, or can be explained by differences in dynamic connectivity between children and adults remains to be further investigated.

## Conclusions

To conclude, using a sample of typical and non-typical children and adolescents, we demonstrated that the direction of the information flow was age dependent for auditory, sensorimotor and cerebellar connections. In addition, the right frontoparietal network had a higher degree of output connections with higher age. These findings contribute to the existing knowledge in the brain development field, and warrant further studies for replication in healthy samples as well as other clinical populations.

## Supporting information

S1 Fig Diagnosis information. Number of diagnoses given to HBN participants that were part of main analysis (N = 1143), where diagnoses are grouped by category. This is based on the final consensus diagnosis given by the lead clinician at the end of participation.
(TIF)

S2 Fig Age distributions within scanning sites. Age distributions within scanning site for the HBN participants that were part of the main analysis (N = 1143), where N = 83 was from the Staten Island site, N = 503 from CBIC and N = 557 from RUBIC/Rutgers scanning site.
(TIF)

S3 Fig Scanner effects on edge-level. Matrices showing the effects of Rutgers scanner (a), and effects of scanner located at Staten Island (b). The analysis was performed in HBN data that had no missing values (N = 1143, 6–17 years, df = 1132). Significant edges following Bonferroni correction are marked as X. The legend shows the 10 RSNs included in the analysis; VO, visual occipital; DMN, default mode; VM, visual medial; VL, visual lateral; FPR, frontoparietal right; FPL, frontoparietal left; SM, sensorimotor; Cer, cerebellum; Au, auditory; Ex, executive control network. The y-axis indicates the sender node, while the x-axis refers to the receiving node. The colors reflect the z-value for the corresponding effects where red indicates a positive association and blue a negative association.
(TIF)

S4 Fig Scanner effects on node-level. Associations on the node level (N = 1143, 6–17 years, df = 1132). a) Out-degree matrix with corresponding effects of covariates scanner in HBN data. B) In-degree matrix with corresponding effects for the same scanner covariates as in panel a). The colors reflect the t-value for the corresponding effect where numbers inside the boxes indicate t-statistic and p-value, and significant effects are marked with a black border following Bonferroni correction (p < 0.05).
(TIF)

S5 Fig Correlation among measurements. Correlation between the covariates; age, sex, mental health, cognitive abilities, tSNR, site and motion included in the HBN model. tSNR and motion were highly correlated as would be expected but none of the other covariates were found to have a high correlation with each other.
(TIF)

S6 Fig Edge-level results after false discovery rate (FDR) correction. Directed connectivity matrices showing significant proportion of edges, and corresponding associations with age, age2, sex, fluid intelligence, pF, pF2, motion and tSNR for HBN (N = 1143) after FDR correction. The colors reflect the z-value for the corresponding associations where red indicates a positive association and blue a negative association. There were two more significant results for age when thresholding using FDR on edge-level, that included directed connectivity from the right frontoparietal to the executive control network, and from the auditory to the cerebellar node.
(TIF)

S7 Fig Effects of data quality on directed functional connectivity. Directed connectivity matrices showing significant proportion of edges, and corresponding associations with age, age2, sex, fluid intelligence, pF, pF2, motion and tSNR for HBN (N = 1028) after exclusion of 10 percent (N = 115) of individuals with the poorest scan quality based on tSNR estimates. The colors reflect the z-value for the corresponding associations where red indicates a positive association and blue a negative association.
(TIF)

S8 Fig Effects of data quality on directed functional connectivity. Directed connectivity matrices showing significant proportion of edges, and corresponding associations with age, age2, sex, fluid intelligence, pF, pF2, motion and tSNR for HBN (N = 1028) after exclusion of 10 percent (N = 115) of individuals with the highest degree of motion. The colors reflect the z-value for the corresponding associations where red indicates a positive association and blue a negative association.
(TIF)

S9 Fig Correlations between IC’s included in this study and Smith’s components. Spatial correlation between the ten RSNs that we included for analysis and the corresponding RSNs from Smith, Fox [59].
(TIF)

S1 Table Z and P_Bonf_ values for effects of age on directed connectivity on the edge-level. (TIF)

S2 Table Z and P_Bonf_ values for effects of FSIQ on directed connectivity on the edge-level. (TIF)

S3 Table Z and P_Bonf_ values for effects of motion on directed connectivity on the edge-level. (TIF)

S4 Table Z and P_Bonf_ values for effects of tsnr on directed connectivity on the edge-level. (TIF)

S5 Table Z and P_Bonf_ values for effects of scanner: Rutgers on directed connectivity on the edge-level. (TIF)

S6 Table Z and P_Bonf_ values for effects of scanner: StatenIsland on directed connectivity on the edge-level. (TIF)
