# Supplementary figures and images for: Functional connectivity directionality between large-scale resting-state networks across typical and non-typical trajectories in children and adolescence
Source: PLoS One. 2022 Dec 1;17(12):e0276221. doi: 10.1371/journal.pone.0276221 (PMC9714732; doi:10.1371/journal.pone.0276221)

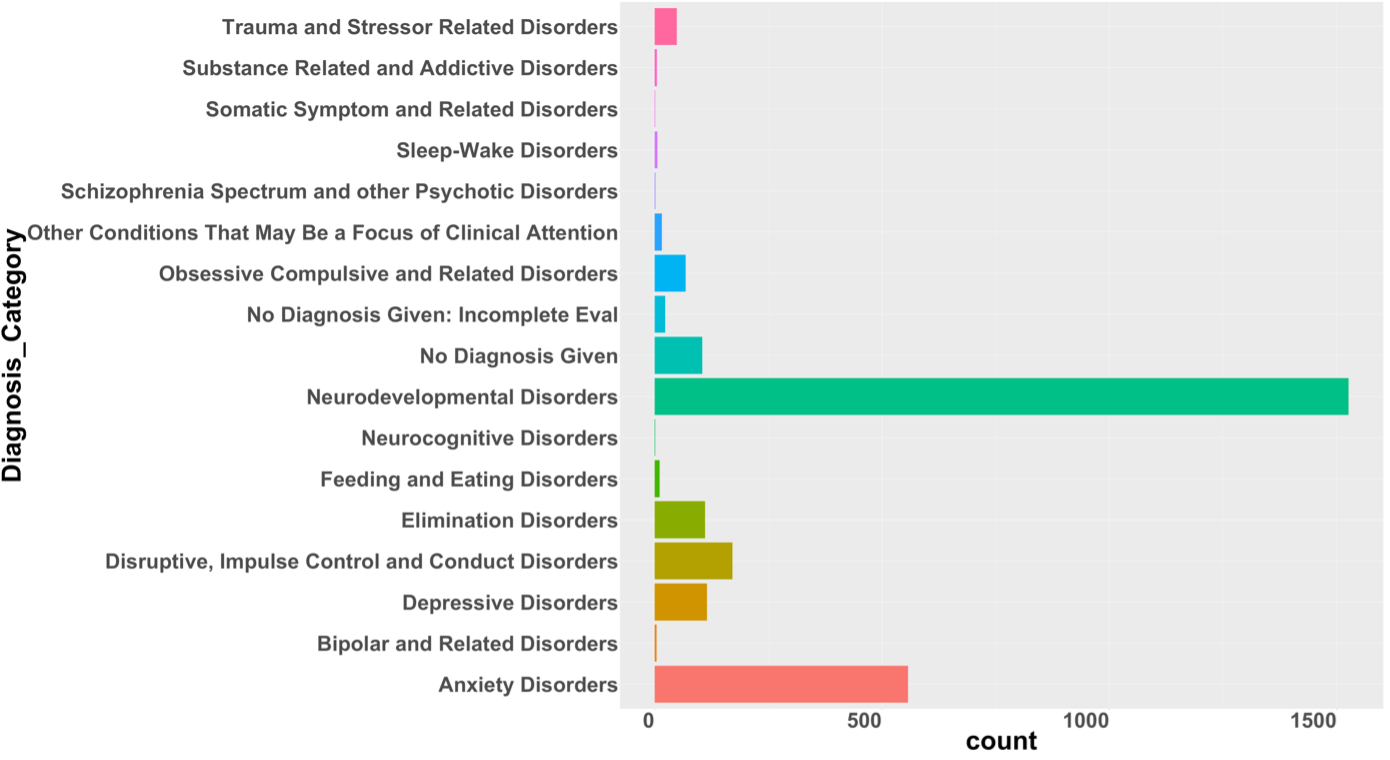

Supplement: S1 Fig — Number of diagnoses given to HBN participants that were part of main analysis (N = 1143), where diagnoses are grouped by category. This is based on the final consensus diagnosis given by the lead clinician at the end of participation. (TIF) [file pone.0276221.s001.tif]

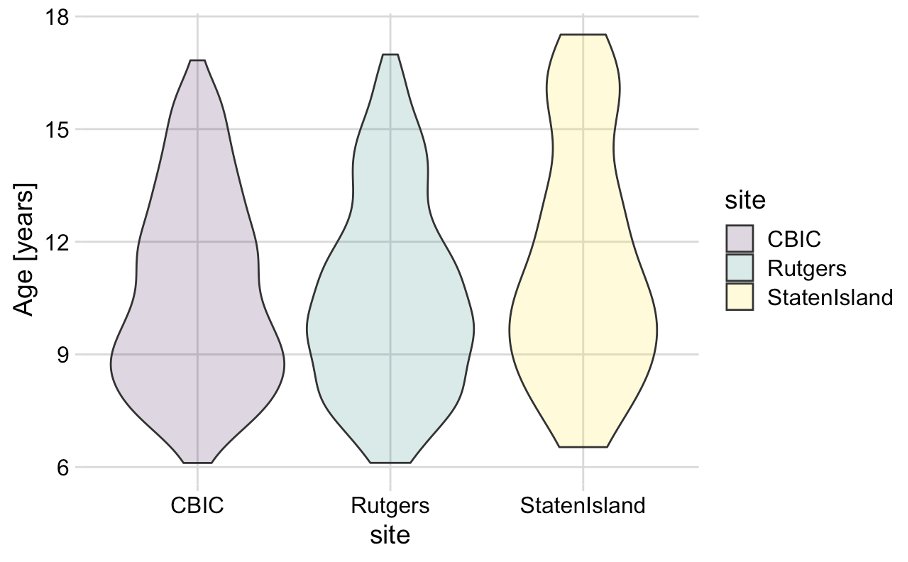

Supplement: S2 Fig — Age distributions within scanning site for the HBN participants that were part of the main analysis (N = 1143), where N = 83 was from the Staten Island site, N = 503 from CBIC and N = 557 from RUBIC/Rutgers scanning site. (TIF) [file pone.0276221.s002.tif]

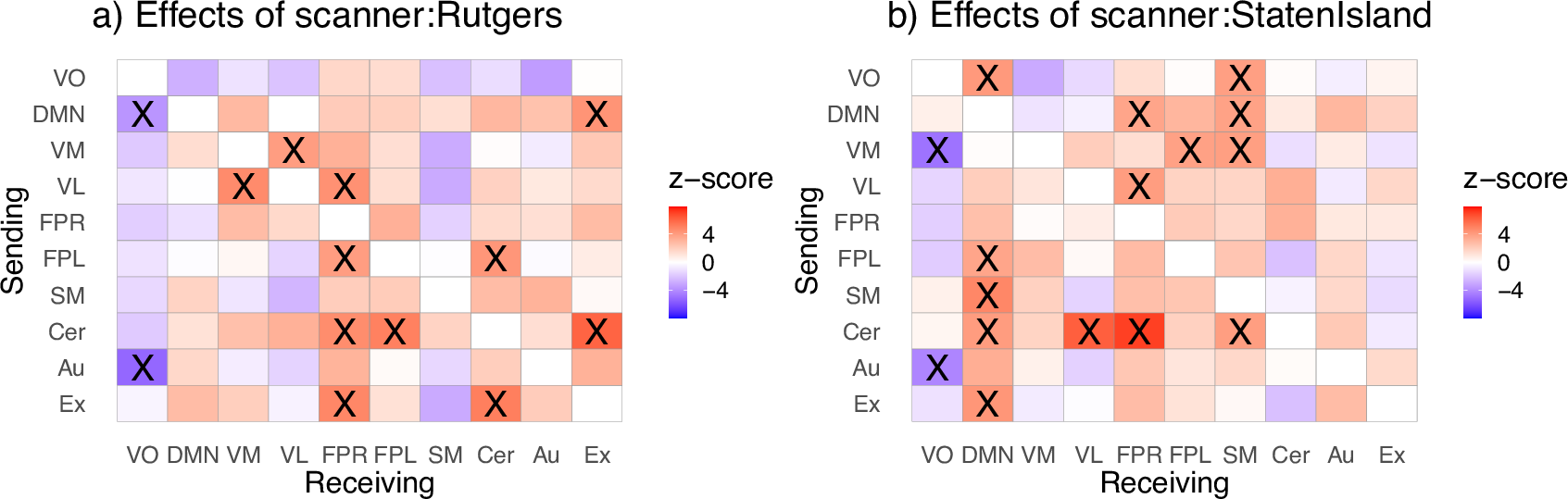

Supplement: S3 Fig — Matrices showing the effects of Rutgers scanner (a), and effects of scanner located at Staten Island (b). The analysis was performed in HBN data that had no missing values (N = 1143, 6–17 years, df = 1132). Significant edges following Bonferroni correction are marked as X. The legend shows the 10 RSNs included in the analysis; VO, visual occipital; DMN, default mode; VM, visual medial; VL, visual lateral; FPR, frontoparietal right; FPL, frontoparietal left; SM, sensorimotor; Cer, cerebellum; Au, auditory; Ex, executive control network. The y-axis indicates the sender node, while the x-axis refers to the receiving node. The colors reflect the z-value for the corresponding effects where red indicates a positive association and blue a negative association. (TIF) [file pone.0276221.s003.tif]

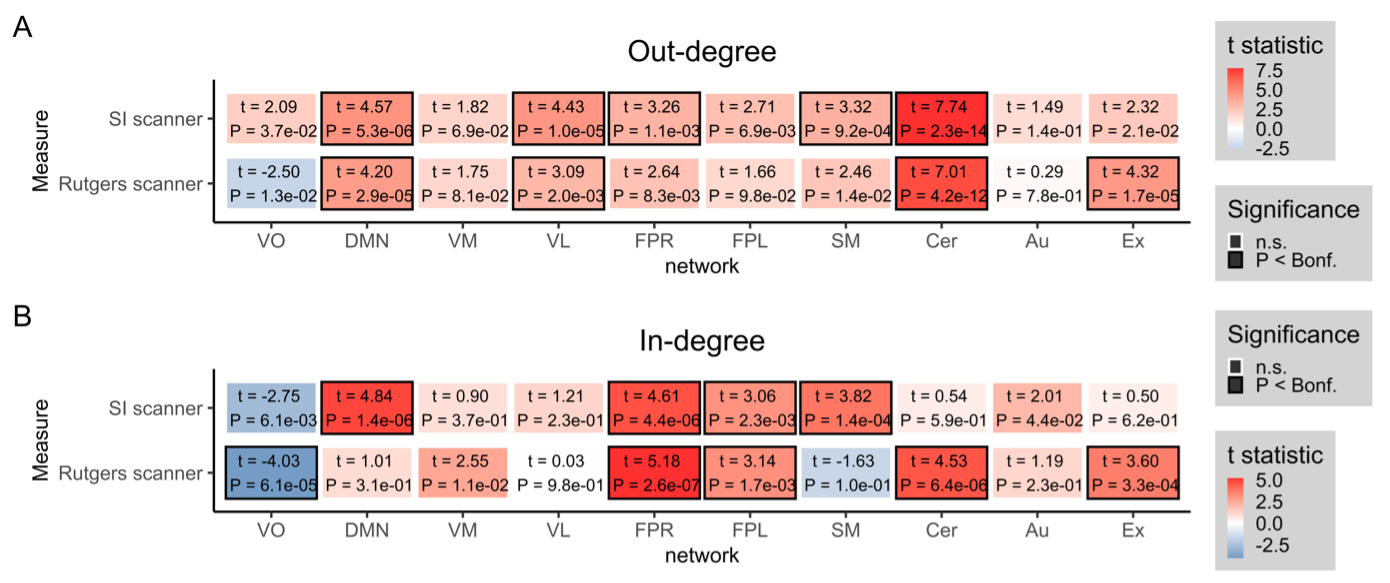

Supplement: S4 Fig — Associations on the node level (N = 1143, 6–17 years, df = 1132). a) Out-degree matrix with corresponding effects of covariates scanner in HBN data. B) In-degree matrix with corresponding effects for the same scanner covariates as in panel a). The colors reflect the t-value for the corresponding effect where numbers inside the boxes indicate t-statistic and p-value, and significant effects are marked with a black border following Bonferroni correction (p < 0.05). (TIF) [file pone.0276221.s004.tif]

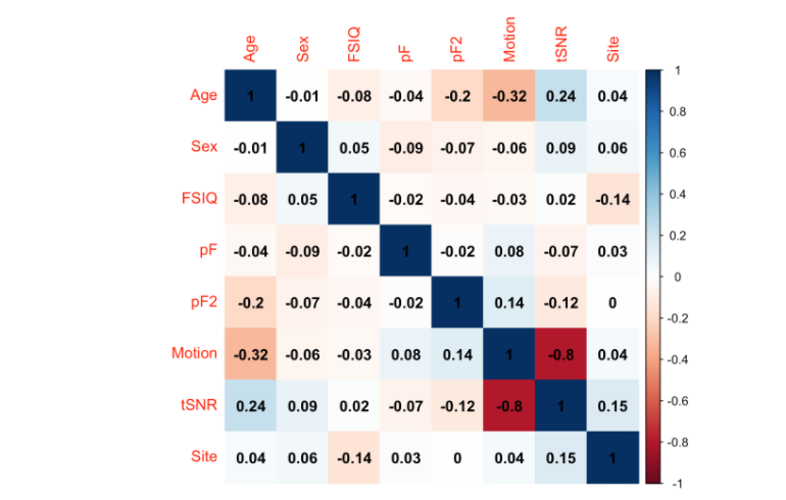

Supplement: S5 Fig — Correlation between the covariates; age, sex, mental health, cognitive abilities, tSNR, site and motion included in the HBN model. tSNR and motion were highly correlated as would be expected but none of the other covariates were found to have a high correlation with each other. (TIF) [file pone.0276221.s005.tif]

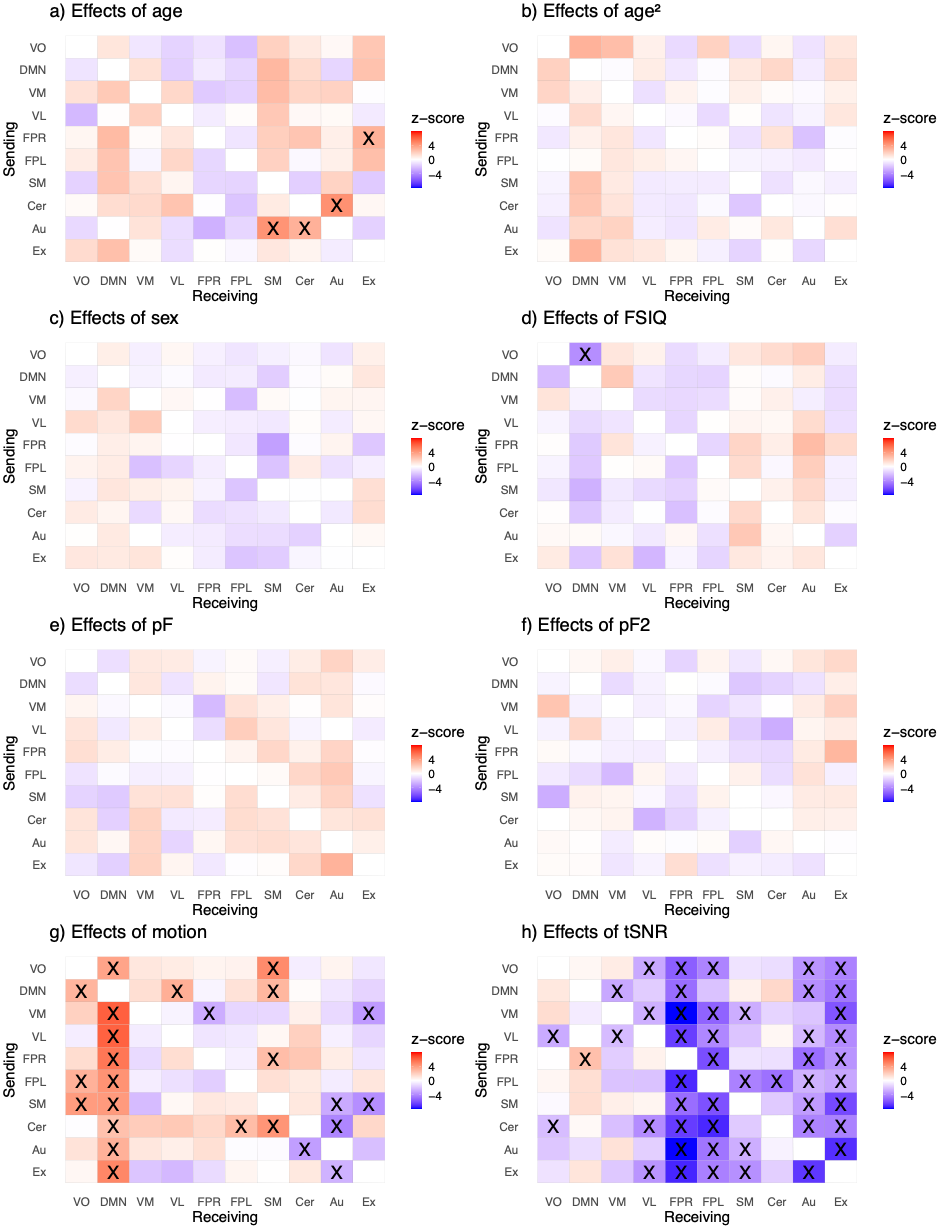

Supplement: S6 Fig — Directed connectivity matrices showing significant proportion of edges, and corresponding associations with age, age2, sex, fluid intelligence, pF, pF2, motion and tSNR for HBN (N = 1143) after FDR correction. The colors reflect the z-value for the corresponding associations where red indicates a positive association and blue a negative association. There were two more significant results for age when thresholding using FDR on edge-level, that included directed connectivity from the right frontoparietal to the executive control network, and from the auditory to the cerebellar node. (TIF) [file pone.0276221.s006.tif]

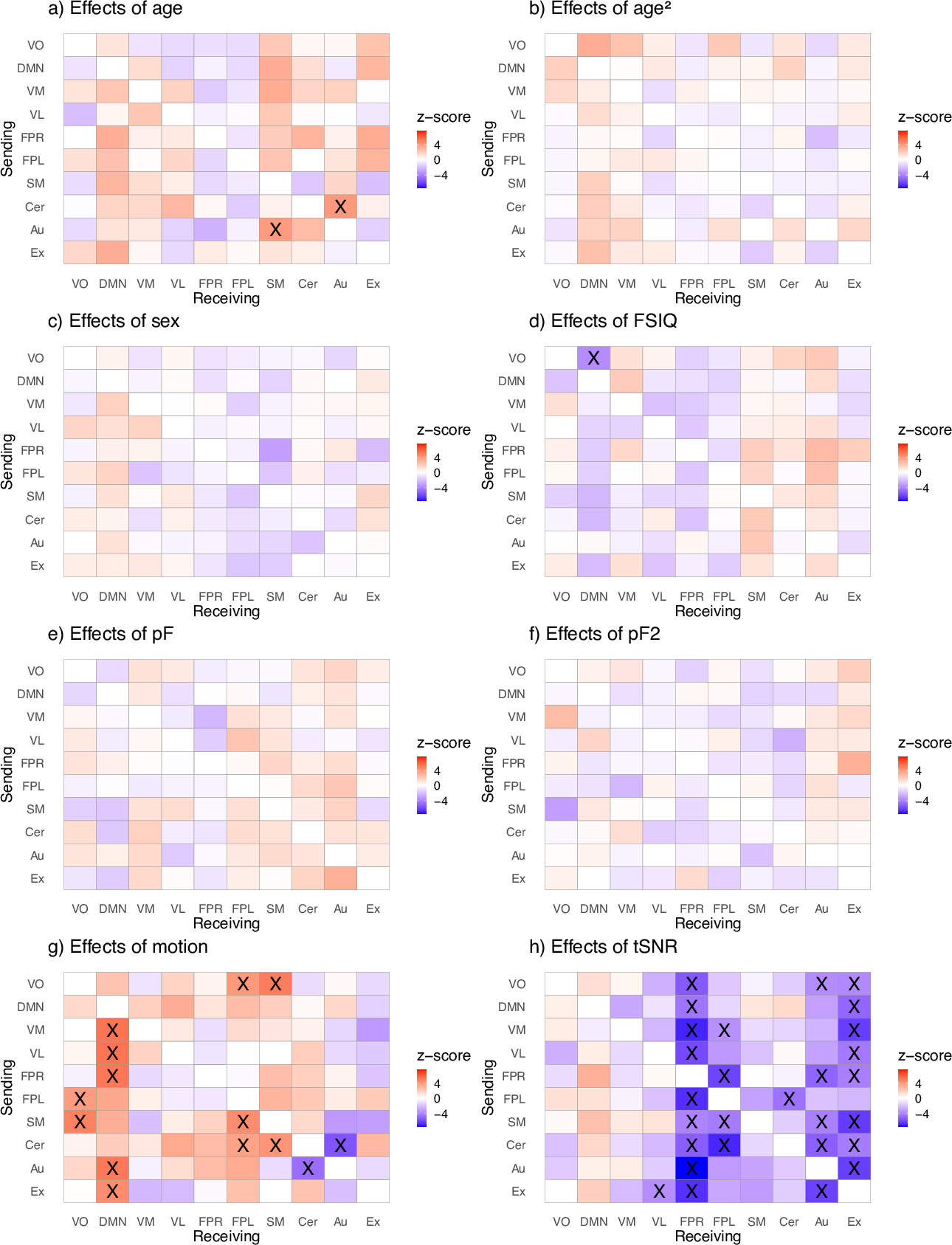

Supplement: S7 Fig — Directed connectivity matrices showing significant proportion of edges, and corresponding associations with age, age2, sex, fluid intelligence, pF, pF2, motion and tSNR for HBN (N = 1028) after exclusion of 10 percent (N = 115) of individuals with the poorest scan quality based on tSNR estimates. The colors reflect the z-value for the corresponding associations where red indicates a positive association and blue a negative association. (TIF) [file pone.0276221.s007.tif]

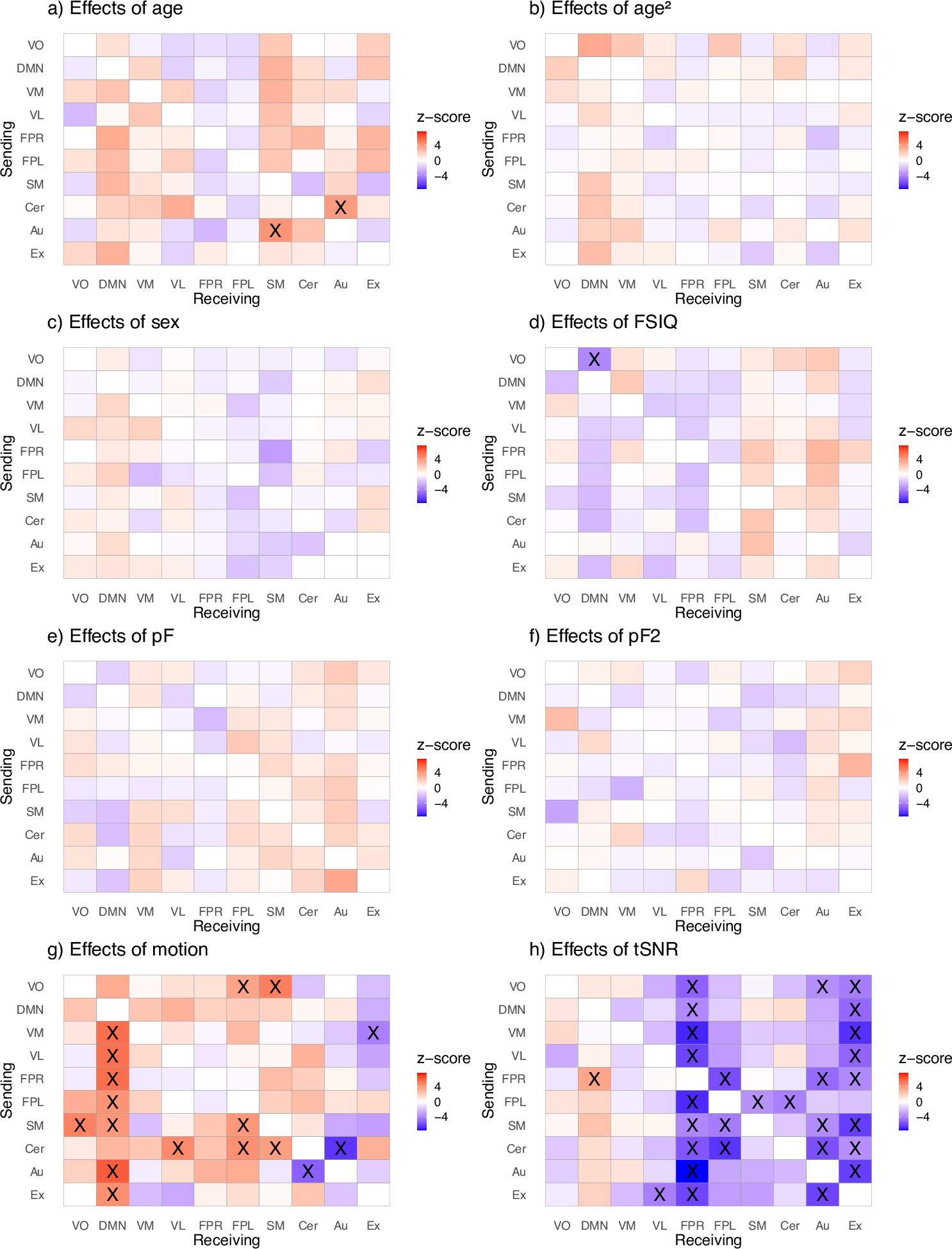

Supplement: S8 Fig — Directed connectivity matrices showing significant proportion of edges, and corresponding associations with age, age2, sex, fluid intelligence, pF, pF2, motion and tSNR for HBN (N = 1028) after exclusion of 10 percent (N = 115) of individuals with the highest degree of motion. The colors reflect the z-value for the corresponding associations where red indicates a positive association and blue a negative association. (TIF) [file pone.0276221.s008.tif]

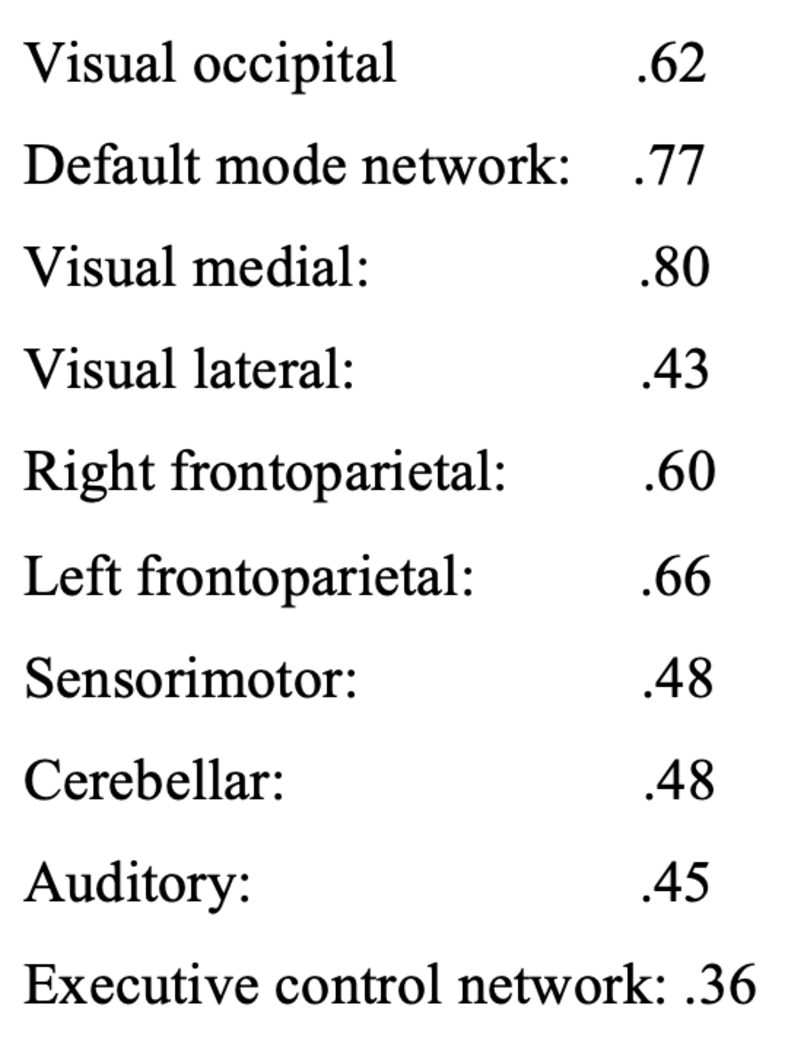

Supplement: S9 Fig — Spatial correlation between the ten RSNs that we included for analysis and the corresponding RSNs from Smith, Fox [59]. (TIF) [file pone.0276221.s009.tif]

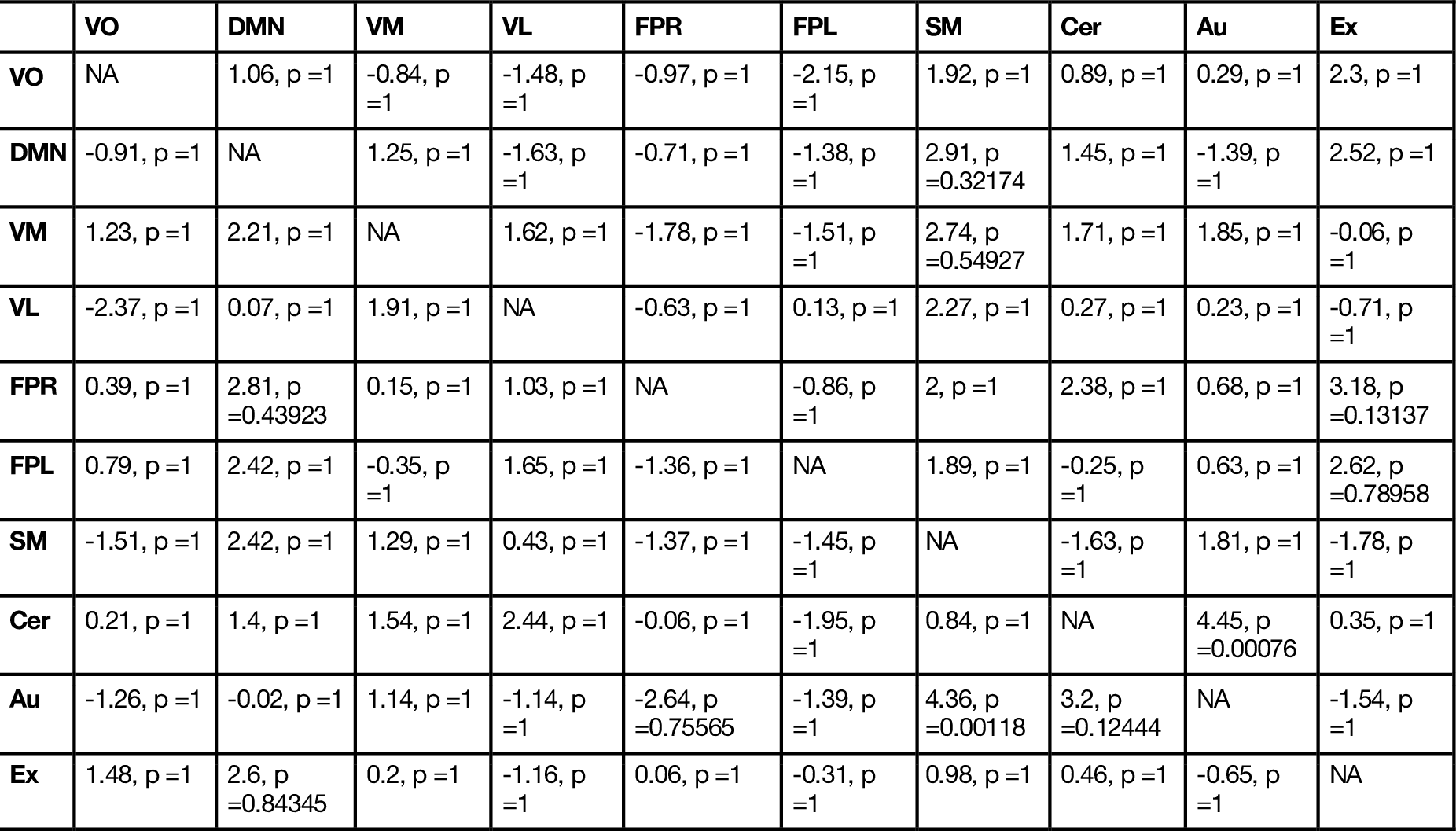

Supplement: S1 Table — (TIF) [file pone.0276221.s010.tif]

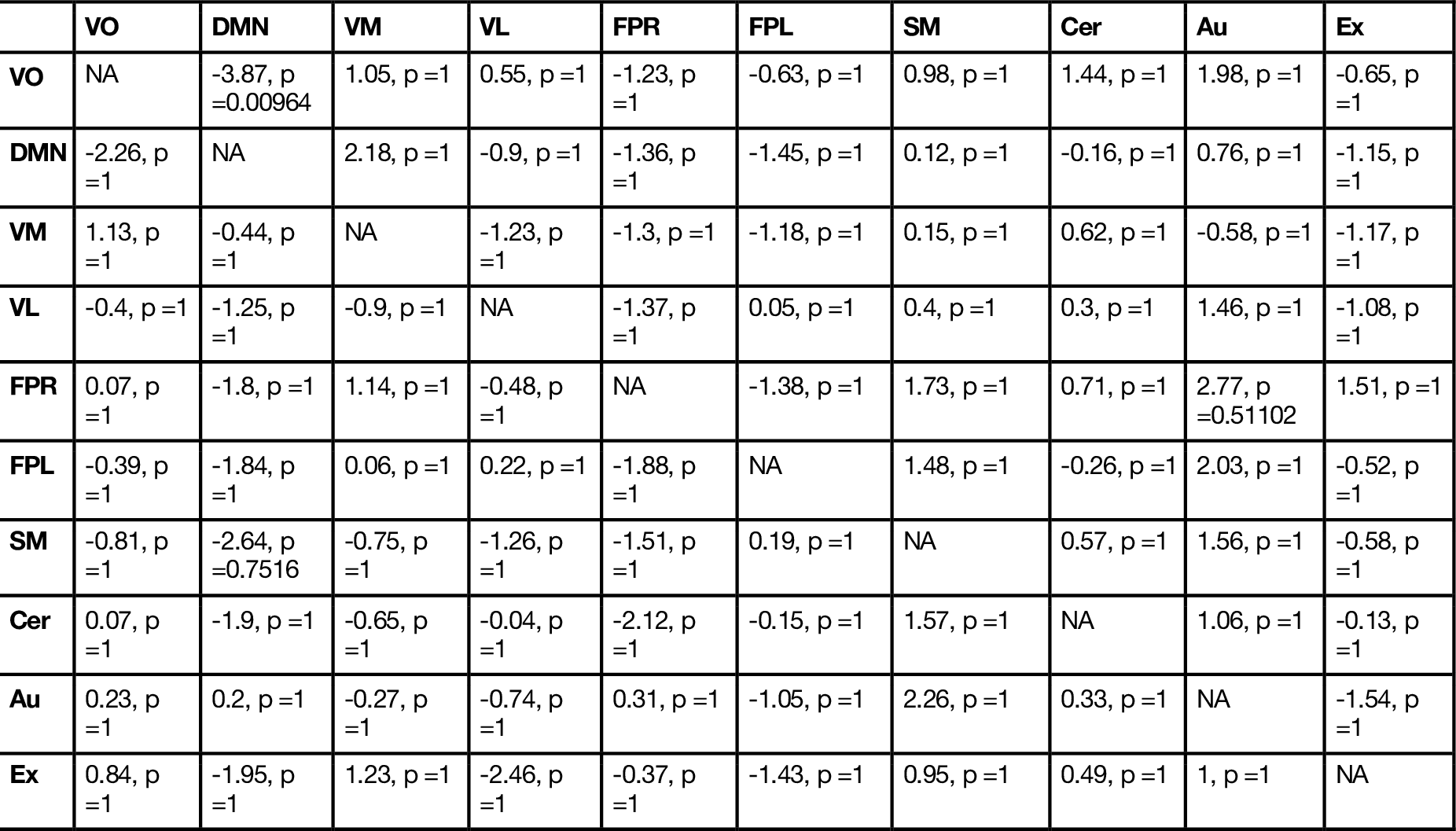

Supplement: S2 Table — (TIF) [file pone.0276221.s011.tif]

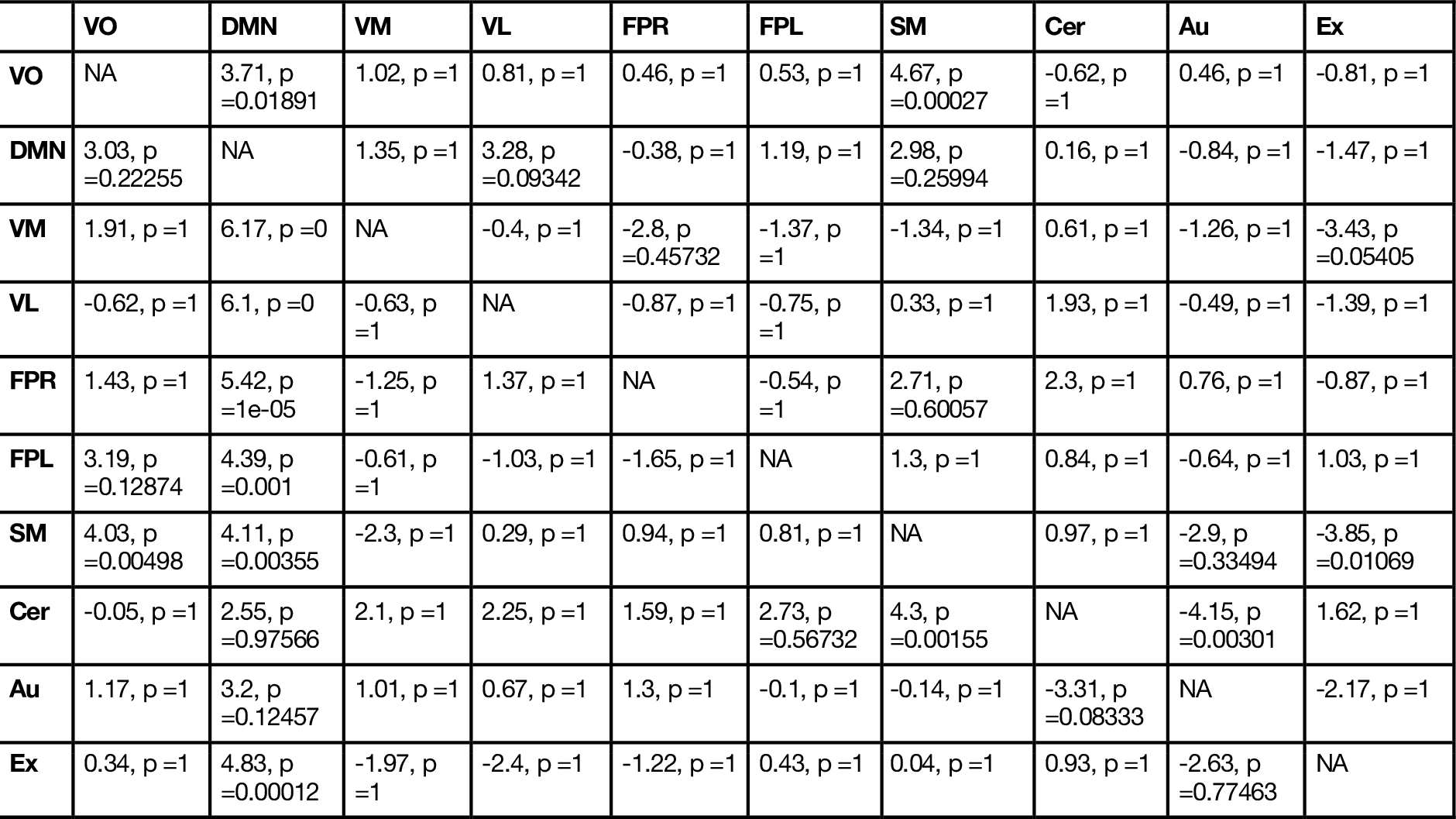

Supplement: S3 Table — (TIF) [file pone.0276221.s012.tif]

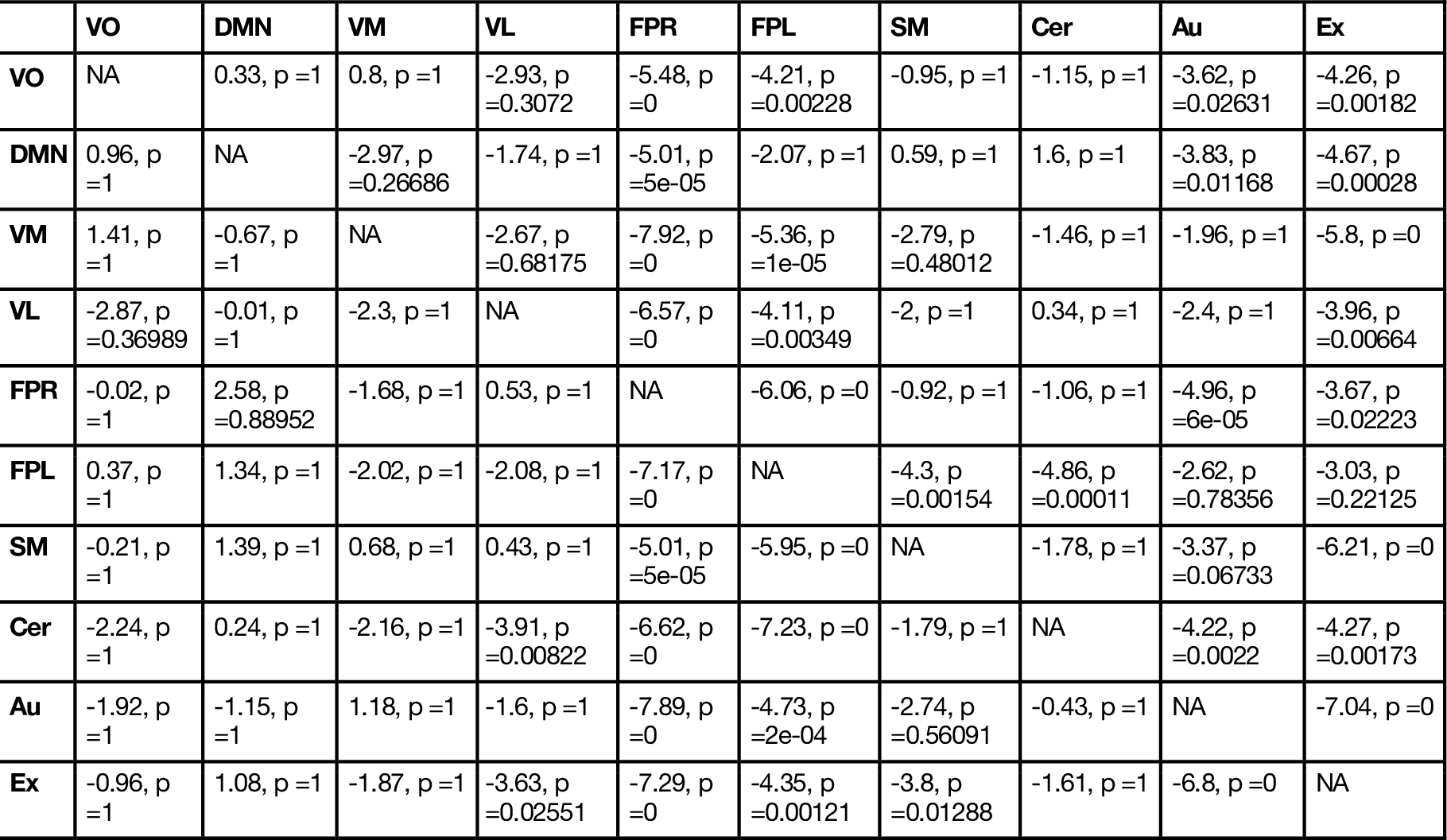

Supplement: S4 Table — (TIF) [file pone.0276221.s013.tif]

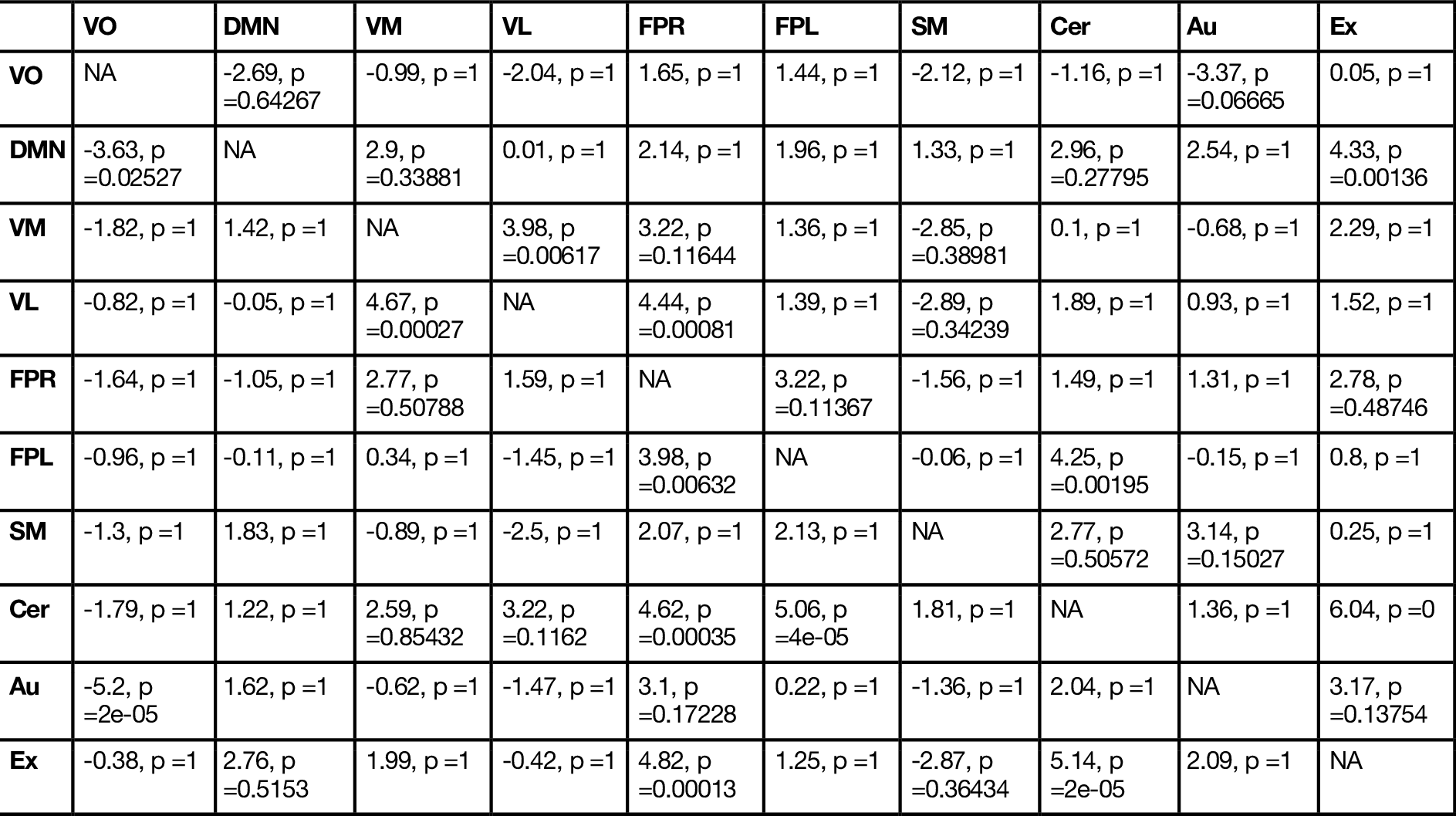

Supplement: S5 Table — (TIF) [file pone.0276221.s014.tif]

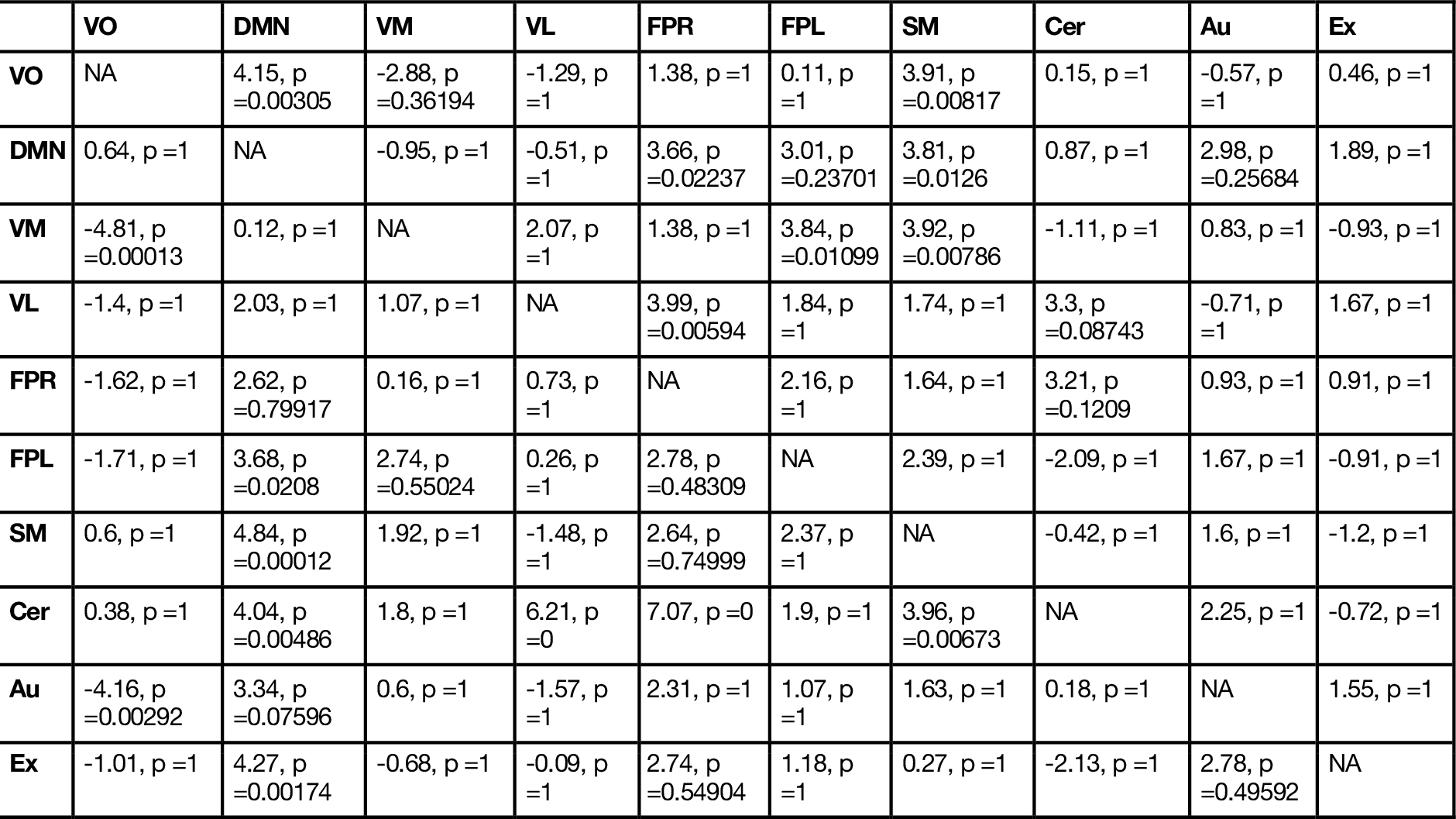

Supplement: S6 Table — (TIF) [file pone.0276221.s015.tif]
